# Book Review of *Alternatives Beyond Psychiatry *by Peter Stastny and Peter Lehmann (Eds)

**DOI:** 10.1186/1747-5341-3-18

**Published:** 2008-07-23

**Authors:** Paul Hammersley

**Affiliations:** 1 Programme Director for Post Graduate Studies in CBT for Psychosis, COPE Initiative, University of Manchester, UK

## Abstract

Peter Stastny and Peter Lehmann's Alternatives beyond Psychiatry offers a comprehensive and up to date account of the alternatives to mainstream psychiatry that are being developed by service consumers and survivors across the world. As psychiatry moves into a new age less dominated by a biomedical paradigm many of the approaches described in this book may be adopted by mainstream health services. This is a hugely readable and accessible book for professionals and consumers alike.

## About the author

Paul Hammersley is the Programme Director for Post Graduate Studies in cognitive behavioural therapy for psychosis at Manchester University's COPE Initiative in The United Kingdom. He is also an active therapist specialising in CBT for individuals experiencing severe psychological problems following traumatic life events. He has been widely published and has lectured extensively. 2006 along with Professor Marius Romme from Holland and The UK Hearing Voices Network, he founded CASL (The Campaign for the Abolition of the Schizophrenia Label.)

## Competing interests

The author declares that they have no competing interests.
